# Timing of Graves’ Hyperthyroidism Management in Pregnant Women: Impact on the Infant Thyroid Volume

**DOI:** 10.3390/nu14091972

**Published:** 2022-05-09

**Authors:** Aleksandra Mikołajczak, Renata Bokiniec

**Affiliations:** 1 Neonatal and Intensive Care Department, Institute of Mother and Child, 01-211 Warsaw, Poland; aamikolajczak@wp.pl; 2 Neonatal and Intensive Care Department, Medical University of Warsaw, Karowa 2, 00-315 Warsaw, Poland

**Keywords:** Graves’ disease, thyroid volume, neonate, ultrasound, antithyroid drugs

## Abstract

The thyroid of the fetus of a mother with Graves’ disease (GD) is affected by the transplacental passage of both antithyroid drugs (ATDs) and thyroid-stimulating hormone receptor antibodies (TRAb). Thyroid hormone imbalances are harmful for the developing brain. This study aimed to evaluate the impact of the duration of antithyroid drug treatment in hyperthyroid pregnant women with GD on infants’ thyroid volume. Twenty-nine neonates born to mothers with GD were divided into two groups depending on the duration of ATDs treatment. The ultrasound thyroid volumes of the infants were measured within the first week of life. Thyroid-stimulating hormone, thyroxine, and TRAb values were recorded. There was no difference between groups in the thyroid hormones’ values. The median thyroid volume for the entire group of neonates with mothers with GD, for the groups of neonates of mothers with long- and short-treated GD, and for the control group were 1.539, 1.816, 1.347 and 1.014 mL, respectively. There were statistically significant differences in the thyroid volume between the GD group and the control group, as well as between the groups of neonates of mothers with long- and short-treated GD (*p* < 0.05). Studies have shown that the longer the duration of ATDs administration to mothers, the greater the thyroid volume of the neonate.

## 1. Introduction

Thyroid hormones play a crucial role in neurodevelopment in fetal and neonatal life as well as in perinatal adaptation [1]. Thyroxine (T4) and triiodothyronine (T3) production begins by the 12th week of gestation, whereas active iodine trapping occurs by the 10th–12th week of gestation. Thyroid function remains at a basal level until mid-gestation, and steadily develops until the 36th week. The production of thyroid hormones is regulated by the thyroid-stimulating hormone (TSH) and is released in response to the regulation of the hypothalamic–pituitary–thyroid (HPT) axis. TSH receptors become responsive to TSH and receptor antibodies (TRAbs) around the 20th week of gestation. Maternal T4 passes through the placenta from the mother to the fetus at approximately 30% of the maternal concentration, simultaneously playing a significant role in the development of the fetal nervous system, particularly before the 12th week of gestation [2,3].

Disturbances of fetal and neonatal thyroid hormone balance pose a risk of damage to the developing brain [4]. Hyperthyroidism in women of childbearing age is most commonly caused by Graves’ disease (GD) [5].

GD is an autoimmune condition caused by the overstimulation of TRAbs [6]. Antibodies may cross the placenta and stimulate the production of thyroid hormones by the fetus, resulting in fetal or neonatal hyperthyroidism. Similar to other autoimmune diseases, GD typically improves during pregnancy, which is associated with increased gestational immune tolerance that aims to prevent the rejection of the fetus [5,6]. Consequently, the risk of neonatal hyperthyroidism is estimated to be very low and affects only approximately 2% of the offspring of mothers with GD [7].

Although the prevalence of GD is reported in approximately 0.2% of pregnant women [8], maternal hyperthyroidism can result in serious adverse fetal and neonatal outcomes due to the transplacental passage of maternal TRAbs [5]. Inadequate control of maternal GD may be associated with adverse effects, including spontaneous abortion, stillbirth, maternal preeclampsia, heart failure, or thyrotoxic storm. Moreover, poorly controlled hyperthyroidism increases the risk of fetal intrauterine growth restriction, fetal death and preterm birth [9,10,11]. Due to its mass and location, the increase in fetal thyroid goiter size might cause dystocia, esophageal, and tracheal compression, resulting in polyhydramnios or asphyxia [12,13].

The identification of hyperthyroidism in neonates, although infrequent, is vitally important because of the potentially life-threatening nature of the condition. The most frequent neonatal clinical features include hyperexcitability, poor weight gain with normal or large appetite, diarrhea, vomiting, staring, goiter, cardiac failure with arrhythmias, advanced bone age, thrombocytopenia, and hepatosplenomegaly. In severe cases, craniosynostosis, microcephaly, and psychomotor issues may occur [9].

Finally, the thyroid status of the fetus/neonate depends on the balance between TRAbs stimulation and the action of antithyroid drugs (ATDs) crossing the placenta, as the ATDs administered to the mother inhibit the activity of the thyroid gland in the fetus as well as in the neonates. Consequently, particular care is required in the management of pregnant women with GD. According to current guidelines and recommendations, the diagnosis of GD during pregnancy is complex. In addition to laboratory tests, an ultrasonographic evaluation of the fetal thyroid should be promptly performed when an elevated level of maternal serum TRAbs is diagnosed or when a mother is treated with ATDs in the third trimester [14]. A regular monitoring and knowledge of the autoimmune mechanisms during pregnancy often enables a reduction in ATD doses, which can prevent the occurrence of hypothyreosis secondary to ATD. We performed ultrasonography to assess neonatal thyroid volume to gain greater insights into the impact of the timing of ATD administration on thyroid function.

The present study aimed to investigate whether different durations of ATD administration to mothers with GD would influence an infant’s ultrasound thyroid volume.

## 2. Materials and Methods

This was an observational, prospective, single-center, population-based control study. The subjects included 29 neonates born to mothers with GD when elevated TRAb serum concentration was observed at least once during pregnancy, or when mothers were treated with ATDs, but TRAb serum concentration was not assessed. The study was performed at the Neonatal and Intensive Care Department of the Medical University of Warsaw at Princess Anna Mazowiecka Hospital (Poland) between 2009 and 2015.

A total of 177 neonates were included in this study: 29 (male: 12, female: 17) from hyperthyroid mothers with GD and 148 term neonates (male: 95, female: 53) as the control group. Neonates born to mothers with GD were divided into two groups depending on the duration of ATD treatment. The characteristics of the study cohort are summarized in Table 1.

The thyroid volume measurement in the control group was performed in 2009. In order to confirm a normal thyroid function in the group of healthy neonates the thyroid hormones and TSH levels were determined in 18 neonates. Apart from those results, the neonates screening tests for congenital hypothyroidism confirmed that examined neonates had normal values of TSH.

Maternal medical history included information concerning the types of thyroid disorders, history of GD, duration of treatment with ATDs, serum level of TRAbs, and substitution of levothyroxine. Perinatal data included gestational age (GA), body weight at birth, sex, z-score of birth weight, Apgar score at 1 min after birth, and method of delivery. The exclusion criteria were syndromic disorders.

All the recruited neonates were born to mothers with GD. The ultrasound thyroid volume of infants was prospectively collected and performed within seven days of life. Thyroid function data concerning serum TSH and FT4 concentrations in neonates were obtained between 4 and 7 days of life.

The study included 29 infants divided into two groups according to the duration of ATD administration to the mothers with GD. Infants were divided into two groups: group 1 (short-treated): those born to a mother treated with ATDs for no longer than up to the second trimester of pregnancy; and group 2 (long-treated): those treated with ATDs in the third trimester of pregnancy.

Mothers with GD were treated with monotherapy, with methimazole (MMI) or with propylthiouracil (PTU). PTU was administered in doses ranging initially from 100 mg to 150 mg and later 25–100 mg, while MMI was administered in doses of 10–15 mg, and later 5 mg, respectively. Generally, PTU was administered in the first trimester, and replaced with MMI for the second and third trimester. One woman was treated with propylhiouracil, and then with levothyroxine in the third trimester. It is worth highlighting that, nowadays, adding levothyroxine to ATDs is not recommended. Another pregnant woman with T3-predominant GD was treated with MMI and propranolol.

The TRAbs level was not always available in pregnant women, though it was elevated in most cases at least once during pregnancy. The TRAbs level ranged from a slightly above normal level to 40 IU/L.

Pregnant women were treated with ATDs to maintain the total thyroxine (TT4) at 1.5 times the upper limit of the non-pregnant reference range or FT4 in the upper limit of the reference range.

Blood samples (1 mL) were collected during routine blood tests and examined in the hospital laboratory to determine serum thyroid hormone levels. The Architect i1000SR (Abbott Diagnostics) test immunofluorescence assays were used to measure TSH and FT4 levels in serum samples.

Ultrasound evaluation of the neonate’s thyroid was performed using a Philips HD XE system (Philips Healthcare, Eidhoven, the Netherlands). The thyroid gland was measured using a linear array transducer with a high-frequency probe L7–15 MHz, or if required, the L11–12 MHz probe was used to measure higher lengths. All infants were scanned by an experienced ultrasonographer (A.M). The thyroid gland volume was calculated using the formula for a prolate ellipsoid, where thyroid volume = length × breadth × depth × π/6 (0.52). The total thyroid volume was calculated as the sum of the volumes of the individual lobes, disregarding the volume of the isthmus, since this is very low in normal neonates [15,16].

The study was approved by the Bioethical Committee of the Medical University of Warsaw, Poland (number KB 47/2008). All caregivers of the study participants were informed about the method and purpose of the study. The recruiting physicians provided them with a leaflet describing the study. Written informed consent was obtained from all parents.

### Statistical Analysis

Statistical analysis was conducted with Python programming language using several packages, such as SciPy, Numpy, statsmodels, and matplotlib. Categorical variables are presented as n (%) and continuous variables as mean ± SD or median (Q1–Q3), depending on the data distribution. Normality of the data was checked with respect to skewness and kurtosis values. Group comparisons were conducted using the chi-square test for categorical variables and Welch’s *t*-test or Mann–Whitney U test for continuous variables, as appropriate. Correlation analysis was performed using Pearson’s correlation coefficient.

## 3. Results

The median (; inter-quartile range, IQR) volumes of the right thyroid lobe, left thyroid lobe, and both lobes combined were 0.745 (0.611; 0.972), 0.749 (0.468; 0.949) and 1.539 (1.169; 1.845) mL, respectively, for the entire group of neonates of mothers with GD; 0.951 (0.726; 1.484), 0.891 (0.690; 1.579), and 1.816 (1.418; 3.136) mL, respectively, for the group of neonates of mothers with long-treated GD; and 0.714 (0.460; 0.870), 0.573 (0.419; 0.842), and 1.347 (0.923; 1.571) mL, respectively, for the group of neonates of mothers with short-treated GD. There were statistically significant differences between the GD group and the control group, as well as between the groups of neonates with mothers who received long- or short-term treatment (*p* < 0.05) (Table 2).

The correlation between the thyroid volume and the TSH value, as well as between the thyroid volume and the FT4 value, was assessed for the entire group of neonates of mothers with GD, and for the group of neonates of mothers with long- and short-treated GD. We did not confirm a statistically significant correlation between the thyroid volume and the TSH value, nor did we find a correlation between the thyroid volume and the FT4 value.

Among the smaller group of mothers treated in the long term, four of the twelve neonates presented with goiter associated with hyperthyroidism with a mean thyroid volume of 3.090, >95 percentile; (min, max: 1.169–5.216) mL. A color flow Doppler examination was performed to reveal global thyroid vascularization. Markers of hyperthyroidism, such as the TRAbs serum concentration, were high in both groups of mothers in the third trimester as well as in their neonates within 4–7 days of life (mothers’ TRAbs: 18.9–40.0, norm < 1.5 IU/L). The TSH serum levels of the neonates were very low (mean TSH = 0.0066 mIU/L).

The TSH concentration in these hyperthyroid neonates was negatively correlated with the thyroid volume (r = −0.613).

The four infants with hyperthyroidism were born earlier (mean gestational age 35 weeks vs. 38 weeks; *p* < 0.05) and had a significantly lower birth weight (mean birth weight 2695 g vs. 3344 g; *p* < 0.05).

The remaining infants in this group presented a different range of hypertrophy of the thyroid gland (max: 2.790 mL, >95 percentile; min: 1.124 mL, 60 percentile).

The results of the thyroid volume, TSH and FT4 of the groups are presented in Table 3.

We did not observe any statistically significant differences in TSH and FT4 levels between the groups (*p* > 0.05) (Table 4).

It should be mentioned that in one investigated infant, the TSH value of 31.0 UI/mL was much higher than in the other infants. This value caused a high standard deviation (SD) of the TSH value, and the SD values in the GD group and in the long-treated group were 5.9 and 9.1, respectively. When this one outlier was excluded, the value of SD was 2.5. Similarly, with regard to the FT4 results, the results of four infants were outlying, which caused a higher value of SD.

## 4. Discussion

The thyroid of the fetus of a mother with GD is affected by the transplacental passages of both ATDs and TRAbs. The administration of ATDs in active GD can result in the development of fetal and neonatal hypothyroidism. Simultaneously, TRAbs tend to stimulate the fetal/neonate thyroid, posing a risk to the development of fetal/neonatal hyperthyroidism [1]. Thyroid function abnormalities can adversely affect fetal and neonate brain development.

Therefore, the aim of this study was to evaluate the impact of the duration of antithyroid drug treatment of hyperthyroid pregnant women with GD on the infant’s thyroid volume.

To our knowledge, no published report has yet investigated the association between ATDs administered to pregnant women and the thyroid volume of infants.

Ultrasound is an imaging tool for assessing the thyroid anatomy, volume, and vascularization of the fetal and neonatal thyroid glands [17,18,19]. It is a useful tool for estimating whether the thyroid gland is normal, small, or large, provided there is access to nomograms for both fetal [20] and neonate populations [21]. Normative data for the fetal thyroid gland were expressed as the diameter or circumference of the gland. The images allowed us to make a differential diagnosis between a hyperthyroid goiter, due to the action of TRAbs antibodies, or a hypothyroid goiter, resulting from antithyroid treatment. An additional tool is color Doppler ultrasound, which reveals vascularization. The latter can aid in distinguishing between hypothyroid and hyperthyroid goiters with the central vascularization characteristic of a hyperthyroid and the peripheral vascularization of the hypothyroid. [17]. Moreover, Huel et al. proposed a diagnostic ultrasound score (vascularization, fetal heart rate, bone maturation, and fetal movements) for assessing fetal goiter [17].

Ultrasound scans should be performed from 20 weeks of gestation in pregnant women with GD treated with ATDs and/or with positive TRAbs.

Advanced bone age, fetal heart rate exceeding 160 bpm, and intrauterine growth restriction are features indicative of cases of fetal hyperthyroidism. Conversely, delayed bone maturation, the slowing of fetal heart rate or decreased fetal movements are associated with fetal hypothyroidism. Invasive examinations, such as cord blood samples collected by funipuncture and amniotic fluid sampling, are generally unnecessary. These procedures should be reserved (and applied) in exceptionally difficult diagnostic situations [7].

Sonography might be helpful to modify the treatment of GD pregnant women with a fetal goiter. It should be mentioned that among ATDs, PTU belongs to drugs with lower teratogenic effects than MMI and should be recommended in the first trimester of pregnancy. The generally preferred MMI should be applied in the second and third trimester one dose daily. PTU is associated with hepatotoxicity [7].

The first finding of our study was that the median (IQR) of the thyroid volume of neonates born to mothers with GD was significantly higher than that of the control group (*p* < 0.05).

**Figure 1:**
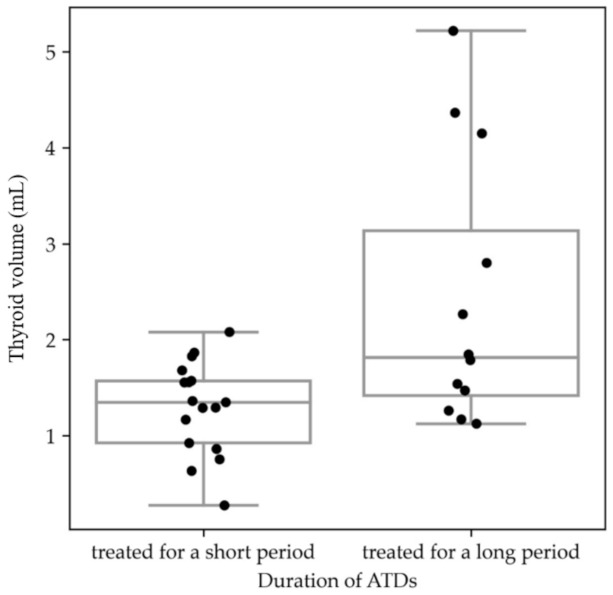
Boxplot: distribution of thyroid volume (mL) presented in two groups of neonates of mothers with Graves’ disease treated in the short and long term with antithyroid drugs. Thyroid volume results of the infants are shown as black dots.

We confirmed statistically significant differences in thyroid ultrasound between the two groups of neonates born to mothers with GD: in the first group of infants, mothers were treated with ATDs no longer than until the second trimester of pregnancy, whereas in the second group, mothers were treated with ATDs in the third trimester of pregnancy. In the latter group, the thyroid volume was higher than that in the group in which the administration of ATDs was discontinued earlier in pregnancy (*p* < 0.05) (Figure 1).

The second finding was the positive relation between thyroid volume and the duration of ATDs administration.

Therefore, our results confirmed the influence of ATDs on the fetal/neonate thyroid. The number of neonates in the group of pregnant women treated until the end of pregnancy was smaller (12 infants) than that in the group not treated in the last trimester (17 infants).

The low number of pregnant women treated in the third trimester may reflect pathophysiological changes in the amelioration of autoimmune diseases during pregnancy.

In our study, infants with neonatal hyperthyroidism (NH) presented with elevated TRAb titers and very low TSH serum levels. Together with thyroid hypervascularization, confirmed by Doppler ultrasound, these are useful markers of NH. According to Banige et al., TSH <0.9 IU/L alone is a good marker of NH in non-autoimmune diseases [22]. Our study highlights that ultrasonography is an important diagnostic tool. In particular, the color Doppler option might be a good predictor of thyroid disturbance.

In our study, infants with NH were born earlier and had a lower birth weight, which is consistent with the findings of Banige et al. [22]. Infants diagnosed with NH in our study were postnatally treated with MMI for 6–8 weeks until the disappearance of TRAbs, which is in agreement with the previous studies [23,24].

Similarly, our study confirmed that color Doppler ultrasound is a useful tool for distinguishing the etiology of goiters with regard to vascularization; the hyperthyroid goiter showed a central vascularization, and the hypothyroid goiter presented with a periphery vascularization [17,25].

Among infants of mothers long treated with ATDs, in one neonate, we observed a high TSH level (31.0 mIU/L), which was associated with a goiter (thyroid volume: 4.36 mL; >95 percentile). This confirmed the appearance of temporary hypothyroidism. Moreover, the goiter caused a deflexed head (hyperextension), and a cesarean section was performed due to high risk of labor dystocia.

From a diagnostic perspective, women with a current or past history of GD should be carefully monitored. The level of TRAbs may increase and remain elevated for months to years in women with a history of ablative therapy (radioiodine or thyroidectomy) [5,6].

According to the current guidelines, it is necessary to investigate the dynamics of TRAbs. In all of these women, the assessment of TRAbs should be performed in the first trimester. Where high TRAb titers (>6 times above the upper limit of normal) are found, TRAbs measurements should be repeated at mid-gestation (18–20 weeks) and late pregnancy (30–34 weeks), if elevated at mid-gestation [5,6,7]. It is necessary to remember that ‘TRAbs’ is a general term used to define antibodies that bind to the TSH receptor. TRAbs can stimulate, block, or be neutral to the TSH receptor, but the occurrence of inhibiting antibodies is exceptional [17]. Currently, only bioassays can differentiate between the actions of TRAbs. Pregnant women should be administered the lowest necessary dose of ATD to maintain TT4 at 1.5 times the upper limit within the non-pregnant reference range or FT4 in the upper limit of the reference range [6].

In the second and third trimester, the autoimmunological disease tends to improve, and the administration of ATDs to the mother is often reduced or even discontinued based on hormone concentrations and TRABs levels, in order to achieve euthyreosis and to prevent fetal hypothyroidism due to ATDs administration. Yet, the presence of TRABs penetrating through the placenta can cause the development of fetal hyperthyroidism, particularly in the second and third trimester, due to the maturation of TSH receptors in the fetal thyroid.

Current guidelines recommend the ultrasound monitoring of the fetus from the 20th week of gestation in pregnant women with elevated TRAbs or treated with ATDs. Differential diagnosis comprises assessing whether the goiter is a hyperthyroid goiter due to the action of TRAbs antibodies or a hypothyroid goiter resulting from antithyroid treatment manifesting as increased central vascularization in the case of a hyperthyroid goiter and decreased peripheral vascularization in the case of a hypothyroid goiter [17].

Persistently elevated TRAbs levels during pregnancy are prognostic of fetal dysfunction, and a high level of TRAbs in the third trimester is a risk factor for the development of neonatal hyperthyroidism [6].

Neonates from mothers testing negative for TRAbs during the second half of gestation and with negative cord blood tests can be discharged and require no further follow-up [25].

Considering that both hyperthyroidism and hypothyroidism may have deleterious effects on the brain, the prompt diagnosis and appropriate treatment of maternal GD during pregnancy may prevent serious consequences in the mother and fetus and, subsequently, in the neonate.

This study has cognitive value as it is the first to compare the size of the thyroid volume in neonates in relation to the duration of ATDs administration to mothers during pregnancy.

This observation also confirms the value of our previously created nomograms for the thyroid volume of neonates.

By demonstrating the impact of the drugs administered to pregnant mothers on the thyroid volume in neonates, we can conclude that an ultrasound assessment of the fetal thyroid can help to guide the treatment of a mother with GD. In doubtful situations, cordocentesis can prove helpful in evaluating the fetal serum concentrations of TSH and FT4 [26].

A limitation of our study is the small number of subjects; nevertheless, GD is a rare condition that improves during pregnancy. Second, this was a single-center study. Third, we did not evaluate the iodine status of pairs: mothers and neonates, but pregnant women in Poland received an iodine supplement according to the Polish Endocrine Society recommendations. Finally, we did not register the expected results with regard to the correlation between neonatal thyroid volume and TSH values, which might have been related to the small size of the sample and the extremely low TSH values common in neonates with hyperthyroidism.

## 5. Conclusions

Studies have shown that the ATD administration to mothers in the third trimester may contribute to hypertrophy of the thyroid gland or even goiter in the offspring.

The results of the present study indicate the necessity to carefully analyze the treatment of pregnant women with ATDs during the last weeks of pregnancy.

A high titer of TRAbs and a very low TSH serum level, together with thyroid hypervascularization confirmed by Doppler ultrasound, are useful markers of neonatal hyperthyroidism.

In summary, ultrasonography might add an important element for assessing the thyroid volume in infants born to GD mothers while color Doppler might also allow, by showing vascularization, hypothyroidism to be distinguished from a hyperthyroid goiter.

## Figures and Tables

**Table 1:** Demographic characteristics of the study cohort.

Characteristics	Graves’ Disease Group *n =* 29	Control Group *n =* 148	*p*	Graves’ Disease Long-Treated Group *n =* 12	Graves’ Disease Short-Treated Group *n =* 17	*p*
Gestation age, weeks, mean (SD)	38 (2.2)	39 (1.2)	NS	37 (2.8)	39 (1.4)	NS
Birth weight, gram, mean (SD)	3344 (617.1)	3446 (450.4)	NS	3118 (599.9)	3443 (564.6) 3503 (594.9)	NS
Sex, male, *n* (%)	12 (41.4)	95 (64.0)	<0.05	7 (58.3)	5 (29.4)	NS
Cesarean section, *n* (%)	18 (62.1)	51 (34.4)	<0.05	7 (58.3)	11 (64.7)	NS
Natural delivery, *n* (%)	11 (37.9)	91 (61.5)	<0.05	5 (41.7)	6 (35.3)	NS
Vaccum extractor, *n* (%)	0	6 (4.1)	NS	0	0	NS
SGA, AGA, LGA						
SGA *n* (%)	0	8 (5.4)	NS	0	0	NS
AGA *n* (%)	22 (75.9)	126 (85.1)	NS	9 (75.0)	13 (76.5)	NS
LGA *n* (%)	7 (24.1)	14 (9.5)	NS	3 (15.0)	4 (23.5)	NS
Apgar score						
>7 points, *n* (%)	27 (93.1)	144 (97.0)	NS	11 (91.2)	14 (82.4)	NS
4–7 points, *n* (%)	2 (6.9)	4 (3.0)	NS	1 (8.8)	3 (17.6)	NS
<4 points, *n* (%)	0	0	NS	0	0	NS

**Table 2:** Thyroid parameters between groups.

		Graves’ Disease Group *n =* 29	Control Group *n =* 148	*p*	Graves’ Disease Long-Treated Group *n =* 12	Graves’ Disease Short-Treated Group *n =* 17	*p*
		Median (IQR)	Median (IQR)		Median (IQR)	Median (IQR)	
Volume (mL)	R	0.745 (0.611; 0.972)	0.502 (0.451; 0.592)	<0.001	0.951 (0.726; 1.484)	0.714 (0.460; 0.870)	0.018
	L	0.749 (0.468; 0.949)	0.511 (0.439; 0.597)	<0.001	0.891 (0.696; 1.579)	0.573 (0.419; 0.842)	0.020
Total volume (mL)		1.539 (1.169; 1.845)	1.014 (0.903; 1.182)	<0.001	1.816 (1.418; 3.136)	1.347 (0.923; 1.571)	0.032

Data are expressed as median and inter-quartile ranges (IQR) for nonparametrical outcomes: R—right; L—left. Abbreviations: mL—milliliter, R—right lobe, L—left lobe, NS—non-significant. The reference ranges of serum FT4 and TSH were 16.7–30.9 pmol/L and 1.7–11.9 mIU/L, respectively.

**Table 3:** Thyroid parameters between groups.

	Gestational Age (Weeks)	Thyroid Volume (mL)	TSH (mIU/L)	FT4 (pmol/L)
L1	40.00	2.80	31.00	21.10
L1	35.00	2.64	2.88	20.50
L1	39.00	4.37	1.89	18.90
L1	38.00	1.47	3.21	19.40
L1	38.00	1.79	-	-
L1	38.00	1.26	1.48	22.78
L1	40.00	1.12	2.36	26.58
L1	37.00	1.54	8.65	25.00
L2	31.00	5.22	0.00	38.78
L2	40.00	1.85	0.02	38.50
L2	36.00	4.15	0.00	64.00
L2	3300	1.17	0.00	47.70
S1	38.00	1.29	2.39	20.60
S1	40.00	2.08	2.35	28.20
S1	37.00	1.58	-	-
S1	37.00	1.29	3.12	20.10
S1	39.00	1.83	-	-
S1	38.00	1.56	1.57	21.59
S1	39.00	0.75	0.67	19.73
S1	39.00	1.36	2.47	20.90
S1	41.00	0.86	-	-
S1	39.00	1.16	1.74	17.30
S1	39.00	1.68	0.55	21.19
S1	39.00	1.86	0.39	23.99
S1	38.00	1.35	0.47	19.39
S1	35.00	0.63	2.24	24.32
S1	37.00	0.27	5.99	20.66
S1	40.00	0.92	1.87	30.00
S1	39.00	1.55	3.69	25.05

L1, L2—infants of mothers with long-treated GD; L2—infants with neonatal hyperthyroidism (NH), treated with ATDs. S1—infants of mothers with short-treated GD.

**Table 4:** Concentrations of FT4 and TSH in neonates assessed between 4 and 7 days of life; comparison between the groups.

	Graves’ Disease Group *n =* 25	Control Group *n =* 19	*p*	Graves’ Disease Long-Treated Group *n =* 10	Graves’ Disease Short-Treated Group *n =* 15	*p*
	Mean (SD) Median (IQR)	Mean (SD) Median (IQR)		Mean (SD) Median (IQR)	Mean (SD) Median (IQR)	
TSH (mIU/L)	3.3 (6.1) 1.9 (0.6; 2.9)	3.1 (1.9) 2.2 (1.9; 3.8)	NS	4.7 (9.1) 1.9 (0.0; 3.0)	2.1 (1.5) 2.1 (0.9; 2.5)	NS
FT4 (pmo/L)	26.3 (10.9) 21.4 (20.1; 27.0)	21.1 (3.9) 20.5 (19.9; 23.3)	NS	31.8 (15.1) 24.7 (20.6; 38.7)	22.3 (3.6) 20.9 (20.1; 24.2)	NS

Data are expressed as mean (SD) and median with inter-quartile ranges (IQR). Abbreviations: TSH—thyroid-stimulating hormone, FT4—free thyroxine, NS—non-significant.

## Data Availability

The data presented in this study are available on request from the corresponding author.
